# Meeting of the Fulton County Medical Society

**Published:** 1914-12

**Authors:** R. R. Daly


### FULTON COUNTY MEDICAL SOCIETY...

### Regular Meeting December 3, 1914.

By R. R. Daly, M. D.

Dr. C. H. Payne presented a case of Complete Left Hydro-Thorax. The patient is a colored woman about 40, married 16 years and in good health until recently. Weight 160. About June she began having hot flashes and sweating. Ascites appeared in August. This was followed by dyspnoea and frequent urination. The abdomen was sore and she went to the college to get treatment for it. She is working every day. The left chest was large and all the intercostal spaces were bulging. Heart dullness and the apex beat were both removed to the right of the sternum. Blood pressure 115-89. Hyaline casts and albument in the urine. 50 c. c. of fluid were withdrawn from the chest and a diagnosis of hydro-thorax due to weak endocardium and nephritis was made.

Dr. S. R. Roberts said it was the first case of complete left hydro-thorax he had seen.

Dr. S. R. Roberts presented a case of Aneurism of Ascending Aorta and Transverse Portion of the Arch. It is one of two cases he has seen where there was a bulging forward of the sterno-clavicular articulation. It pulls the sterno-cleido mastoid forward on that side and turns the head to the left. The patient is a negress, age 39. Blood pressure left arm 120, right 110. There is specific history, pupils are ovoid and the left is larger of the two. The pulse tracing shows force and volume fair but irregular. She has a trophic disturbance in a swelling of the lower lip. She complained in 1913 and 1914 of constant neuralgia in the third rib in front and extending clear to the roots of her hair; the pain is dull, constant and heavy. Later on she had a feeling of pins and needles over both molar regions as if they were numb and frozen. There is no sensation in the right cheek, and no sensation in the left cheek, upper lips and eyelide. The area of pulsation is 1 1-2 inches from the median line below the right clavicle. There is a systolic thrill followed by a dyastolic "tug" felt only during expiration. There is no trachial "tug," but deep pressure above the clavicle shows the thrill. There is a bruit all through systole. The Mitral sounds are clear. The veins in the right neck and sternum are engorged. She cannot lie on either side because of the throbbing, but all signs are increased when she is on the right side. Her kneejerk is exaggerated and she has migraine and sposs before her eyes. She is taking mixed treatment. The fluoroscope shows the action of the tumor.

Dr. E. C. Thrash said that he had posted such cases as this and he doesn't doubt but that there is completer erosion of the sternum and deep erosion in the vertebral bodies. He doubts if the facial disturbance is due to the aneurism directly. It is probably leutic cephalo malaria.

Dr. G. M. Niles said that in making routine fluoroscope examinations of cases, it is remarkable what unsuspected misplacements of thoracic viscera are found. This is also true of the stomach and it is very graphic.

Dr. J. M. Campbell said that an interesting feature of the case is the pain on the right side. It might be due to a pulling on the phrenic nerve and nerves of the cervical plexus. The anaesthesia on the left side is due to disturbance of the fifth nerve, of course, but the connection is not clear.

Dr. H. R. Donaldson asked how long she had had syphilis and what the treatment of the trouble was.

Dr. Roberts closed by saying that if there were traction on the phrenic nerve, there would probably be more gastric disturbance such as hiccoughing. She has some slight exophthalmus and thus the cervical ganglia are involved. In answer to Donaldson, he said that she had a lewd history but had never been treated for syphilis. The present treatment is mixed. The prognosis is of course very grave. He thanked Dr. Niles for assistance and courtesy in the x-Ray work.

Dr. Theodore Toepel read a paper on "Physical Training in the Treatment of Nervous Diseases." It appears elsewhere in the Journal.

Dr. Hansell Crenshaw said that physical training in Tabes is important because some patients who could not walk have been trained to go up and down steps freely. There are two ways of going at it. One is to train the eye to take the place of the spinal sensory paths. This requires that the patient should always watch his step. The other is to make them practice blindfold in the hope of opening up some paths that have not been destroyed and which hitherto have been used for something else.

It is remarkable what can be done in that way. The detail is to begin with the patient lying in bed and train him to sit up after he has learned to move his legs lying down. After this the sense of station is tried and he learns to stand without swaying. Then walking is begun and this is either by marking tracks for him to see or doing it blindfold, according as there are nervous indication. In early cases, good results are obtained.

Dr. Howard Bucknell read a paper on "Milk Therapy in Nutritional Disturbances." It appears elsewhere in the Journal.

Dr. C. E. Boynton said that the paper strikes the keynote of successful infant raising by artificial food. The time has come when drugs are almost discarded in the care of infants and proper diet takes their place.

Dr. W. N. Adkins said that when any baby was once removed from the mother's breast, the best the doctor can do is to imitate the mother's milk and he is fortunate if he succeeds early. The milk from the breast is usually too rich rather than too poor. The breasts in the same mother differ. One may have 5% of fats and the other only 2%. This variation is due in part to the amount of use they get. The one nursed the more is the richer as a rule.

Dr. S. A. Visanski said that while in the winter we never hear of "winter complaints," we have "summer complaints" as a common term. The reason may be in the food that the cows get as they go to fresh pastures and eat things that give them, themselves, diarrhoeas.

Dr. Dunbar Roy read a paper, "A Review of the Advances in Ophthalmology for the Year 1914."

Dr. Howard Bucknell said it was pleasing to have a resume of events in any line and that he was greatly interested in this one. He had noted in the British Journals that corneal ulcers were being treated by ethyl hydrocuprein and that the success was great.

Dr. R. M. Nelson said that in 1910 he had had a case of tremulous iris after spontaneous absorption of cataract.

Dr. W. N. Adkins described a case of brain abscess that came to his attention rather strangely. He was called to the house to see a child of three for some trouble and he noticed a girl of fifteen who appeared dull and listless and was said to be cross at times. The next day she had sore throat and mumbled as she talked.

Her pulse was slow and irregular, temperature normal. There was no pain. She had what appeared to be follicular tonsilitis and a culture was taken, she lapsed into unconsciousness and then quietly spoke to the doctor. Counsel was called and the diagnosis of toxaemia was made without denoting the source until it was discovered that she had otitis media and had suffered with it a long time. The next morning there was vomiting, protrusion of the tongue and bending back of the head. The pupils were irregular and did not react to light. Difficult breathing was following by cessation of breathing. The pulse remained good for ten minutes, but she died in spite of all efforts to continue the breathing.

Drs. Nelson, Rhodes and Sage Hardin all agreed that it must have been a case of cerebral abscess that suddenly increased in size and pressure or ruptured into the Pia.

At the Regular Meeting of the Society, held December 17, 1914, the election of officers resulted as follows: President, S. R. Roberts; Vice-President, W. A. Selman; Secretary, Montague Boyd; Treasurer, A. H. Lindorme; Member of the Board of Censors, J. D. Thomson.

